# Anti-Inflammatory Effects of BoNT/A Against Complete Freund’s Adjuvant-Induced Arthritis Pain in Rats: Transcriptome Analysis

**DOI:** 10.3389/fphar.2021.735075

**Published:** 2021-11-05

**Authors:** Xinhe Li, Yinshuang Ye, Wenwen Zhou, Qilin Shi, Lin Wang, Tieshan Li

**Affiliations:** ^1^ Department of Rehabilitation Medicine, The Affiliated Hospital of Qingdao University, Qingdao, China; ^2^ Department of Rehabilitation Medicine, Qingdao West Coast New District People’s Hospital, Qingdao, China

**Keywords:** BoNT/A, anti-inflammatory, arthritis, pain, transcriptome analysis, differentially expressed genes

## Abstract

Arthritis is the most common cause to lead to chronic pain. Botulinum toxin type A (BoNT/A) has been widely used to treat chronic pain. In our previous study, we confirmed the anti-inflammatory and antinociceptive effects of BoNT/A in the Complete Freund’s Adjuvant (CFA)-induced arthritis model, but the underlying anti-inflammatory mechanism was not fully elucidated. The purpose of this study was to investigate the anti-inflammatory effects and mechanisms of BoNT/A on arthritis using transcriptomic analysis. The BoNT/A was injected into the rat ankle joint on day 21 after CFA injection. The von Frey and hot plate tests were applied to assess the pain-related behaviors at different time points. Five days after BoNT/A treatment, gene expression profiling in dorsal root ganglion (DRG) was performed using RNA sequencing (RNA-seq). The differentially expressed genes (DEGs) were analyzed by various tools. The mechanical allodynia and thermal hyperalgesia were significantly reversed after BoNT/A injection. RNA-seq revealed 97 DEGs between the CFA group and Sham group; these DEGs were enriched inflammatory response, IL-17 signaling pathway, etc. There are 71 DEGs between the CFA+BoNT/A group and the CFA group; these DEGs related to response to peptide, PI3K-Akt signaling pathway, ECM–receptor interactions, etc. Three key genes were significantly decreased after CFA-induced arthritis pain, while BoNT/A increased the expression of these genes. The identification of S100A9, S100A8, and MMP8 genes can provide new therapeutic targets for arthritis pain and affect the signaling pathway to play an anti-inflammatory role after the treatment of BoNT/A.

## 1 Introduction

Arthritis is a progressive disease, characterized by chronic pain, joint destruction, and decreased patient function (Bergman, 2006). Patients with arthritis often suffer from chronic pain leading to severe disabilities and a major burden for individuals and society (Walsh et al., 2015). Chronic pain has a significantly negative impact on quality of life, and this effect increases with the duration of pain. Approaches traditionally used for refractory arthritis pain including medication, intra-articular treatments, topical treatments, and physical treatments had limited efficacy and were at risk of toxicity (Singh, 2013). Therefore, it is important to explore more effective therapeutic options.

Botulinum toxin type A (BoNT/A), a potent neurotoxin, which was used only to treat the hyperactive muscular disorders in clinically effective doses, has been widely used in the treatment of chronic pain recently (Rivera Día et al., 2014). Many years ago, studies of the radiolabeled BoNT/A have found that BoNT/A could be transported retrogradely along axons *via* microtubule trajectories to the central nervous system. Later ultrastructural autoradiographic studies also showed that BoNT/A is retrogradely transported within the axonal compartment of peripherally injected regions. Some studies suggested that the retrograde BoNT/A may release local neurotransmitter by SNAP-25 cleavage to play an antinociceptive effect (Dressler, 2013; Matak and Lackovic, 2014). Previous studies have found that BoNT/A retrogradely transported into the DRG and the expression for IL-1β and TNF-α was reduced in DRG; these results confirmed the anti-inflammatory and antinociceptive effects of BoNT/A in the CFA-induced arthritis model (Fan et al., 2017; Wang et al., 2017). Although the mechanisms of action of BoNT/A have been extensively researched, its precise effects of anti-inflammatory remain unclear.

The current study aimed to investigate the key genes and explore the anti-inflammatory effect of BoNT/A on the CFA-induced arthritis rat model using transcriptome analysis and RNA-seq technology.

## 2 Materials and Methods

### 2.1 Animals

Twenty-seven adult, lab male Sprague–Dawley (SD) rats (180–220 g) were randomly divided into three groups (Sham, CFA, and CFA+BoNT/A group) of nine rats each. All rats were housed in a standard laboratory environment (22 ± 2°C room temperature, 12-h light/dark cycle with lights) with six animals per cage, fed according to a standard rat diet and free water. The housing conditions and experimental procedures were in accordance with the protocols approved by the Bioethics Committee of Qingdao University. The procedures were approved by the Institutional Animal Care and Use Committee and performed in accordance with the NIH guidelines for the care and use of laboratory animals and the International Association for the Study of Pain.

### 2.2 Drug

Monoarthritis (ankle arthritis) was induced as previously reported (Butler et al., 1992). After being anesthetized with 5% isoflurane in oxygen, the rats were placed in a prone position and injected with 50 μl of CFA (Sigma, United States) into the left ankle joint cavity as the CFA group. The control group was injected with saline as Sham group. After 21 days, the CFA model was set up. After the rats were anesthetized with isoflurane, 20 μl of BoNT/A (Allergan Pharmaceuticals Ireland) diluted to 3 U with saline (3 U BoNT/A/20 μl) or 20 μl of saline was also injected into the left ankle joint cavity in the Sham, CFA, and CFA+BoNT/A groups.

### 2.3 Behavioral Tests

#### 2.3.1 Mechanical Allodynia

Paw withdrawal threshold (PWT) in rat with arthritis was measured by using a series of calibrated nylon von Frey hair (Stoelting, Wood Dale, IL, United States), ranging from 0.4 to 15.0 g, according to the method described by Chaplan et al. (1994) to evaluate the mechanical allodynia. Before the test, all rats were placed in transparent plastic cages covered with a wire mesh floor for approximately 20 min to acclimate. The von Frey hairs were passed through the wire at the bottom of the cage, and the midplantar surface of the hind paw was stimulated by the up–down method. The test always started with 2 g of von Frey hair. The time interval between consecutive hair administrations was at least 5 min. Five trials were carried out on the bilateral hind paws. The positive response to stimulation was the rapid withdrawal or licking of the paw.

#### 2.3.2 Thermal Hyperalgesia

Thermal hypersensitivity was measured using a hot plate as described (Yu et al., 2008). All rats were placed on a hot plate to acclimate for 5 min 24 h before the experiment. Briefly, the rats were placed on a hot plate (55 ± 0.5°C) aimed at the adequate plantar surface of the hind paw. The time when the rats lifted or licked their hind paws for the first time was recorded as the paw withdrawal latency (PWL). After the reaction, the rat was promptly taken out of the plate. Three results were recorded in each rat with 15 min interval between tests. The final result was the PWL average of the three tests.

### 2.4 Immunofluorescent Staining of cl-SNAP-25 (Botulinum Toxin Type A Markers) in Dorsal Root Ganglion

On the fifth day after BoNT/A injection, the three groups of rats (*n* = 3/group) were deeply anesthetized. Then, they were perfused with 0.9% saline and 4% paraformaldehyde (pH 7.4) in sequence. The left DRG was removed and placed in perfusion fixative (4°C) for 24 h. After fixation, paraffin embedding was performed, and 8-μm-thick sections were cut along the long axis of the DRG and mounted on 3-aminopropyl-triethoxysilane-coated glass slide. Sections were sealed with phosphate-buffered saline (PBS) containing 3% donkey serum albumin and 0.3% Triton X-100, and then incubated overnight with primary antibody, which was mouse anti-rat cl-SNAP-25 (1:500, MyBioSource, United States). After washing with PBS, DRG sections were incubated with donkey anti-mouse IgG (1:1,000 dilution Invitrogen) labeled with fluorescein isothiocyanate at 37°C for 40 min to determine the immunoreactivity of Cl-SNAP-25. Images were captured on a Leica DMIRB fluorescence microscope at a magnification of 20x. The immunofluorescent staining of protein expression was quantified and analyzed by ImageJ.

### 2.5 RNA Isolation, Library Preparation, and Sequencing

Five days after BoNT/A treatment, the left L4 DRG of the rats were extracted from the Sham group, the CFA group, and the CFA+BoNT/A group (*n* = 3/group). Next, the total RNA of each sample was extracted with TriPure isolation reagent according to the instructions of the kit (Roche), and then genomic DNA was digested in the column in accordance with specific protocols. Thereafter, NanoDrop ND-1000 (Thermo Scientific, Wilmington, DE, United States) was used to measure RNA quality and quantity, and RNA integrity was assessed using the RNA Nano 6000 Assay kit and the Bioanalyzer 2100 System (Agilent, Santa Clara, CA, United States). Total RNA was used as input material for the RNA sample preparations. Briefly, mRNA was purified from total RNA by using poly-T oligo-attached magnetic beads. Fragmentation was carried out using divalent cations under elevated temperature in First Strand Synthesis Reaction Buffer (5×). First-strand cDNA was synthesized using a random hexamer primer and M-MuLV Reverse Transcriptase, and then RNaseH was used to degrade the RNA. Second-strand cDNA synthesis was subsequently performed using DNA Polymerase I and dNTP. Remaining overhangs were converted into blunt ends *via* exonuclease/polymerase activities. After adenylation of 3’ ends of DNA fragments, Adaptor with a hairpin loop structure was ligated to prepare for hybridization. In order to select cDNA fragments of preferentially 200 bp in length, the library fragments were purified with AMPure XP system (Beckman Coulter, Beverly, United States). After PCR amplification, the PCR product was purified by AMPure XP beads, and the library was finally obtained. In order to ensure the quality of the library, the library needs to be tested. After the construction of the library, the library was initially quantified by Qubit2.0 Fluorometer and then diluted to 1.5 ng/μl, and the insert size of the library is detected by Agilent 2100 Bioanalyzer. After insert size meets the expectation, qRT-PCR is used to accurately quantify the effective concentration of the library (the effective concentration of the library is higher than 2 nM) to ensure the quality of the library. After the library is qualified, the different libraries are pooled according to the effective concentration and the target amount of data off the machine and then sequenced by the Illumina NovaSeq 6000. The end reading of 100 bp pairing is generated. The basic principle of sequencing is to synthesize and sequence at the same time (Sequencing by Synthesis). Four fluorescently labeled dNTP, DNA polymerase, and splice primers were added to the sequenced flowcell and amplified. When the sequence cluster extends the complementary chain, each dNTP labeled by fluorescence can release the corresponding fluorescence. The sequencer captures the fluorescence signal and converts the optical signal into the sequencing peak by computer software, so as to obtain the sequence information of the fragment to be tested.

### 2.6 Reads Mapping and Identification of Differentially Expressed Genes

The image data measured by the high-throughput sequencer are converted into sequence data (reads) by CASAVA base recognition. Raw data (raw reads) of fastq format were firstly processed through in-house perl scripts. In this step, clean data (clean reads) were obtained by removing reads containing adapter, reads containing N base, and low-quality reads from raw data. At the same time, Q20, Q30, and GC content of the clean data were calculated. All the downstream analyses were based on the clean data with high quality. HISAT2 (v2.0.4; parameters: -p 8 –phred64 –sensitive -I 1 -X 1000) was employed to map the clean reads to the rat reference sequences. Then, clean reads were mapped to the reference transcript using Bowtie2 (v2.2.5; parameters: -q –phred64 –sensitive –dpad 0 –gbar 99999999 –mp 1 ,1 –np 1 –score-min L,0, -0.1 - p 16 -k 200), and the gene expression values were calculated by RSEM (v1.3.1; parameters: default). Differential expression analysis of three groups was performed using the DESeq2 R package (1.20.0). DESeq2 provides statistical routines for determining differential expression in digital gene expression data using a model based on the negative binomial distribution. The resulting *p*-values were adjusted using Benjamini and Hochberg’s approach for controlling the false discovery rate. |log2(foldchange)| >2 and adjusted *p*-value <0.05 were set as the threshold for significantly differential expression.

### 2.7 Functional Enrichment Analysis

Functional enrichment analysis of DEGs was performed using KEGG and Metascape. KEGG analysis was obtained from KEGG, and biological processes were performed by Metascape.

### 2.8 Protein–Protein Interaction Network Analysis of Differentially Expressed Genes

Protein–protein interaction (PPI) network analysis was performed using the online database STRING to predict PPIs. In addition, the PPI network was constructed and visualized using Cytoscape V3.8.2 software. MCODE was used to conduct cluster analysis on the gene network to determine key PPI network modules.

### 2.9 Total RNA Extraction and RT-PCR Analysis

Five days after BoNT/A treatment, total RNA was extracted from the DRG of the rats in each group (*n* = 3/group) using the TriPure Isolation Reagent according to the instructions of the kit (Roche). RNA purity was determined by Quantus Fluorometer. First, the total template RNA was reverse transcribed into complementary DNA (cDNA). Then, the SYBR PrimeScript PLUS RTPCR kit was mixed with the cDNA samples for qPCR cycling in the AriaMX HRM analysis. GAPDH was used as the reference gene to normalize cycle threshold (Ct) data, and the comparative Ct method was used to calculate each sample.

### 2.10 Statistical Analysis

Throughout the research process, a statistically significant difference analysis (*p* < 0.05) was performed using GraphPad Prism 8 (GraphPad Software, CA, United States). All of the data are shown as mean ± standard deviation (SD). Two-way repeated measure analysis of variance (RMANOVA) was used to compare the results of pain behavior test (PWT and PWL) in each group. Immunofluorescence staining and RT-PCR analysis were performed to analyze the differences between the groups by one-way ANOVA.

## 3 Results

### 3.1 Effects of Botulinum Toxin Type A on Pain Behavior Following Complete Freund’s Adjuvant Injection

**FIGURE 1:**
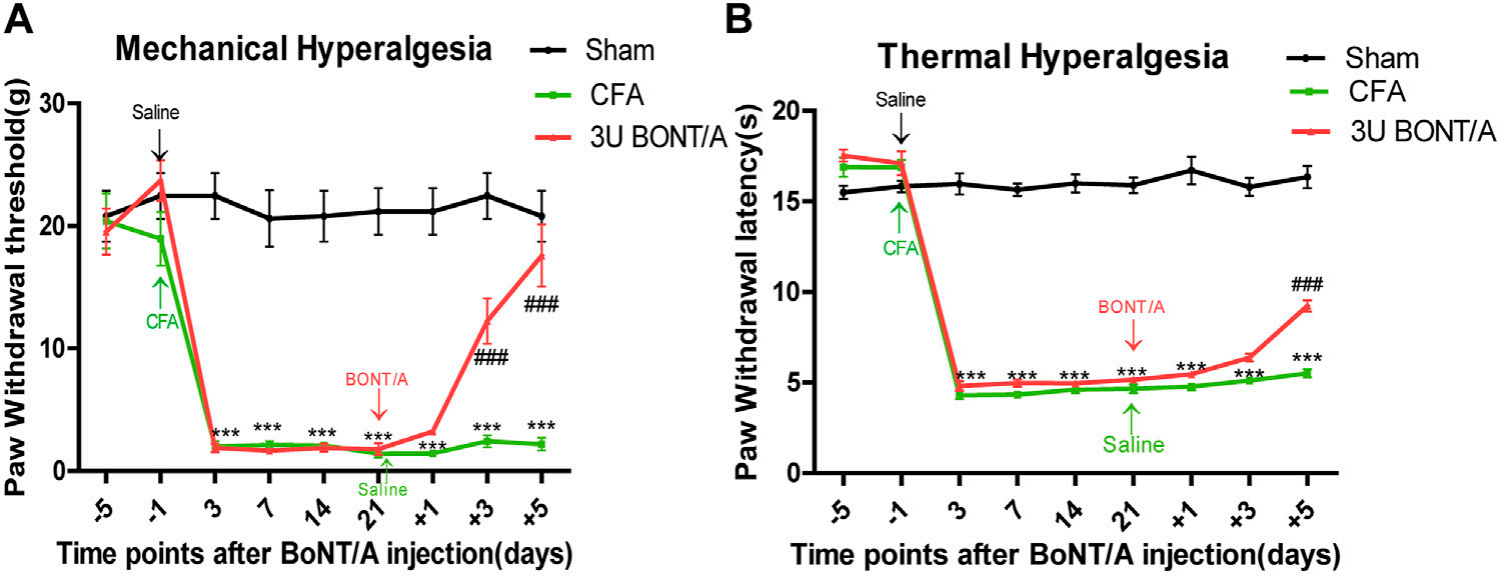
**(A)** Mechanical allodynia presented by paw withdrawal threshold (PWT). **(B)** Thermal hyperalgesia presented by paw withdrawal latency (PWL). The mechanical allodynia and thermal hyperalgesia were significant decreased after CFA injection. After BoNT/A application, PWT significantly increased at doses of 3 U at 5 days, and PWL also displayed similar results (***p < 0.001 represents comparison of CFA with the sham group, ###*p* < 0.001 represents comparison of 3 U CFA+BoNT/A with the CFA group).

All animals developed prolonged mechanical allodynia after CFA injection. During the entire observation period, PWT of mechanical stimulation was significantly lower than that of the Sham group (Figure 1A). Similarly, PWL of thermal stimulation was distinctly shorter than that of the Sham group (Figure 1B). The PWT of CFA+BoNT/A group on mechanical stimulation increased significantly at 5 days after BoNT/A injection. Similarly, the PWL to thermal stimulation was also significantly reversed by the injection of BoNT/A.

### 3.2 Immunofluorescence Analysis of cl-SNAP-25 in Dorsal Root Ganglion

**FIGURE 2:**
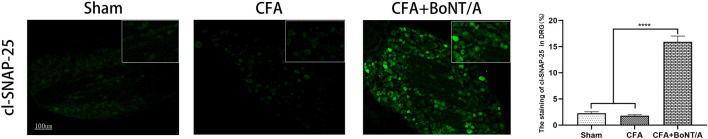
Immunofluorescent staining of cl-SNAP-25 (BoNT/A markers) in DRG at 5 days after BoNT/A injection. Scale bar: 100 μm. (****p < 0.0001, CFA+BoNT/A group vs. Sham group; ****p < 0.0001, CFA+BoNT/A group vs. CFA group; *p* > 0.05, CFA group vs. Sham group).

The expression of cl-SNAP-25 in DRG was analyzed to determine whether retrograde transport of BoNT/A could partially explain the observed effect of anti-inflammatory pain. As reported by Restani et al., it is reasonable to use the cl-SNAP-25 as a marker for BoNT/A. Therefore, the anti-cleaved SNAP-25 antibody was used to mark the BoNT/A action at distant sites. The results showed typical examples of immunofluorescence image taken from ipsilateral dorsal root ganglion (Figure 2). The expression of cl-SNAP-25 could hardly be discovered in the Sham group injected with saline and the CFA group. Conversely, the expression of cl-SNAP-25 was obviously detected after injection of BoNT/A.

### 3.3 Transcriptome Profile of Complete Freund’s Adjuvant and Botulinum Toxin Type A-Treated Rats Generated by RNA-Seq

To determine the potential mechanism of BoNT/A’s anti-inflammatory effect, mRNA was respectively extracted from the Sham, CFA, and CFA+BoNT/A treatment groups, and then transcriptome analysis was performed using RNA-seq. The analysis results showed that 97 DEGs were found between the CFA group and the Sham group. Among them, CFA induced the upregulation of 29 DEGs and downregulation of 68 DEGs. Similarly, a total of 71 DEGs were found between the CFA+ BoNT/A group and the CFA group, of which 52 genes were upregulated and 19 genes were downregulated.

### 3.4 Identification of Key Genes of Complete Freund’s Adjuvant-Induced Arthritis Pain in Rats

**FIGURE 3:**
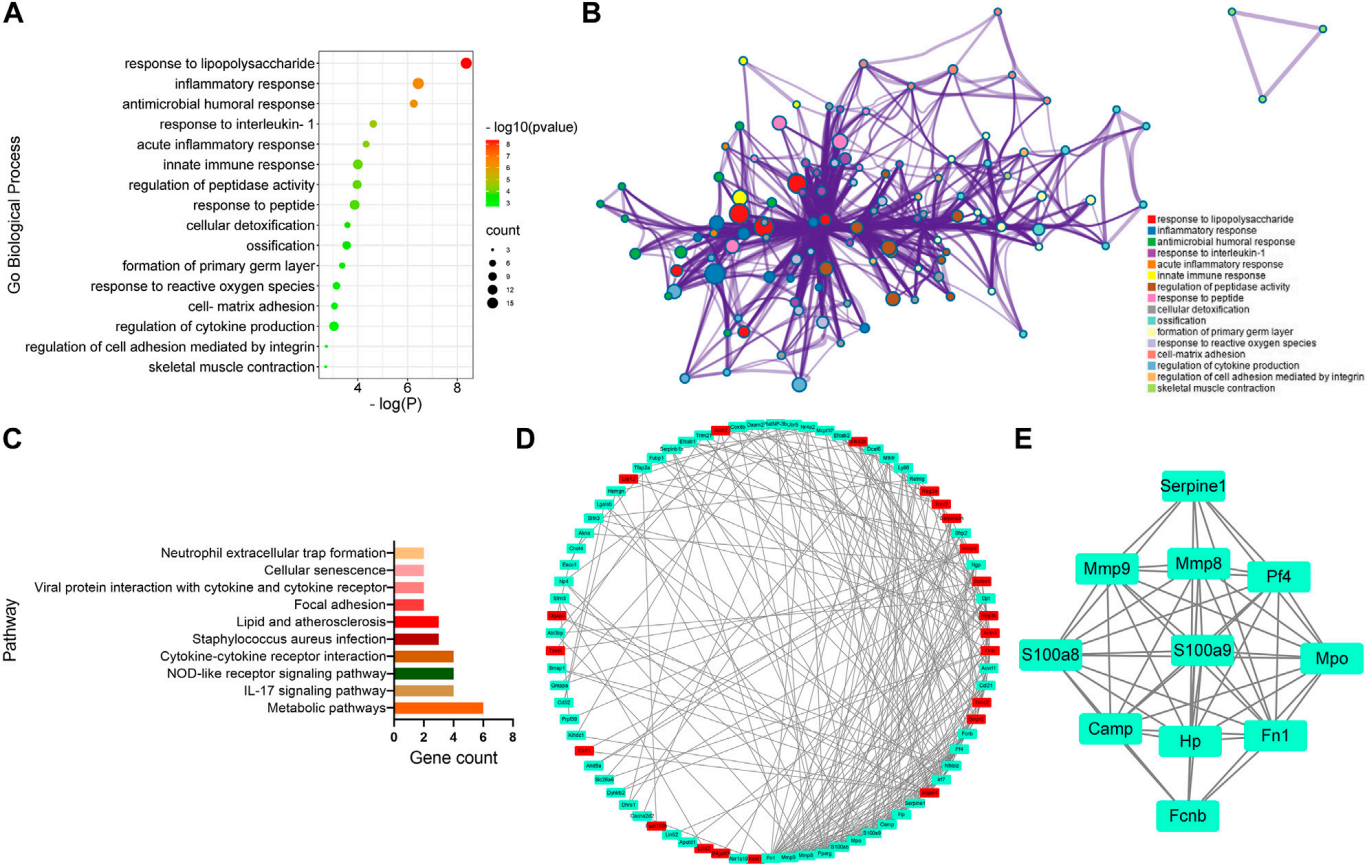
GO, KEGG, and protein–protein interaction (PPI) network analysis of DEGs between the CFA group and Sham group. **(A,B)** GO Biological Process enrichment of DEGs. **(C)** KEGG pathway enrichment of DEGs. **(D)** PPI network of DEGs. **(E)** A cluster was obtained by MCODE in Cytoscape.

To further analyze the genes associated with CFA-induced arthritis pain, 16 different GO terms enrichment analyses were performed on 97 DEGs between the CFA group and the Sham group. All terms were listed, several of which were associated with response to lipopolysaccharide and inflammatory response (Figures 3A,B). In addition, the study also performed pathway analysis on the screened DEGs and matched them to the physiological processes involved in CFA-induced arthritis pain in rats using the KEGG pathway analysis. CFA affected some signaling pathways, for example, metabolic pathways and IL-17 signaling pathway (Figure 3C). We obtained a PPI network that contains 78 nodes and 262 edges. Among 97 DEGs between the CFA group and the Sham group, 21 did not form molecular networks with other molecules. The nodes were used to represent genes and edges to indicate interactions between genes. Upregulated genes were marked in red, and downregulated genes were labeled in cyan (Figure 3D). A cluster including 11 genes was obtained by MCODE in Cytoscape. We discovered these genes (SERPINE1, MMP9, MMP8, PF4, S100A8, S100A9, MPO, CAMP, HP, FN1, and FCNB) encoding proteins about inflammatory response, and these genes might be the key genes of CFA-induced arthritis pain in rats (Figure 3E).

### 3.5 Identification of Potential Genes Involved in the Anti-Inflammatory Effect of Botulinum Toxin Type A Against Complete Freund’s Adjuvant-Induced Arthritis Pain in Rats

**FIGURE 4:**
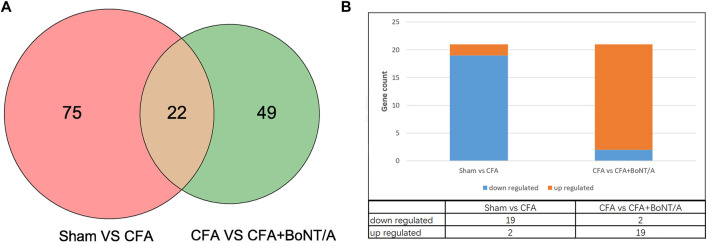
**(A)** Venn diagram showing the overlap of 22 genes between the CFA group vs. Sham group and CFA+BoNT/A group vs. CFA group. **(B)** There were 19 upregulated and 2 downregulated genes between the CFA group vs. Sham group and CFA+BoNT/A group vs. CFA group.

**FIGURE 5:**
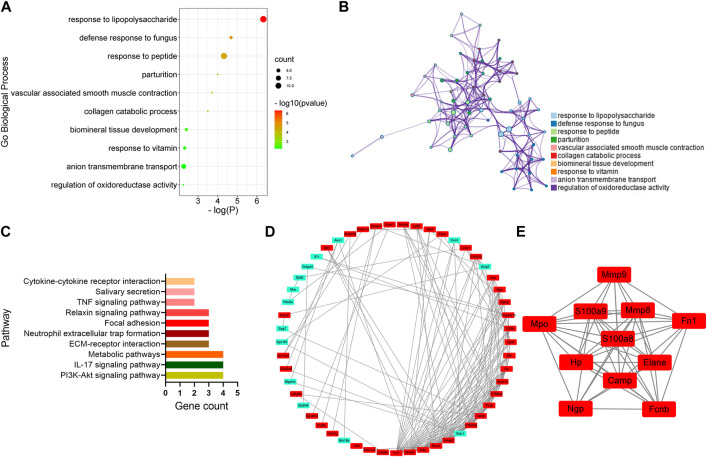
GO, KEGG, and protein–protein interaction (PPI) network analysis of DEGs between the CFA+BoNT/A group vs. CFA group. **(A,B)** GO Biological Process enrichment of DEGs. **(C)** KEGG pathway enrichment of DEGs. **(D)** PPI network of DEGs. **(E)** A cluster was obtained by MCODE in Cytoscape.

To identify the key genes involved in the analgesic effect of BoNT/A on arthritis, this study analyzed the CFA group vs. the Sham group and the CFA+ BoNT/A group vs. the CFA group. Twenty-two overlapping genes were found (Figure 4A). Furthermore, among the 21 DEGs, 19 genes were upregulated and two genes were downregulated, which were compared between the Sham group and the CFA group. However, the expression levels of these 21 different genes were reversed between the CFA+ BoNT/A group and the CFA group (Figure 4B). The study also performed GO and KEGG analysis on DEGs between the CFA+ BoNT/A group and the CFA group. The results indicated that the mechanism of the anti-inflammatory effect of BoNT/A against CFA-induced arthritis pain involves the reactions to lipopolysaccharides and peptides (Figures 5A,B) and their related signaling pathways such as PI3K-Akt signaling pathway and IL-17 signaling pathway (Figure 5C). A PPI network was obtained that contains 53 nodes and 163 edges. Among the 71 genes, 18 were not involved in the formation of the molecular network. The nodes were used to represent genes and edges to indicate interactions between genes. Upregulated genes are marked in red, and downregulated genes are labeled in cyan (Figure 5D). We gained a cluster including 11 genes by MCODE in Cytoscape. We discovered these genes (MMP9, MPO, S100A9, MMP8, FN1, HP, S100A8, ELANE, CAMP, NGP, and FCNB) encoding proteins mostly about chronic inflammatory response, and these genes might be involved in the anti-inflammatory effect of BoNT/A against arthritis pain (Figure 5E).

### 3.6 Validating the Expression of Key Genes by qRT-PCR

**FIGURE 6:**
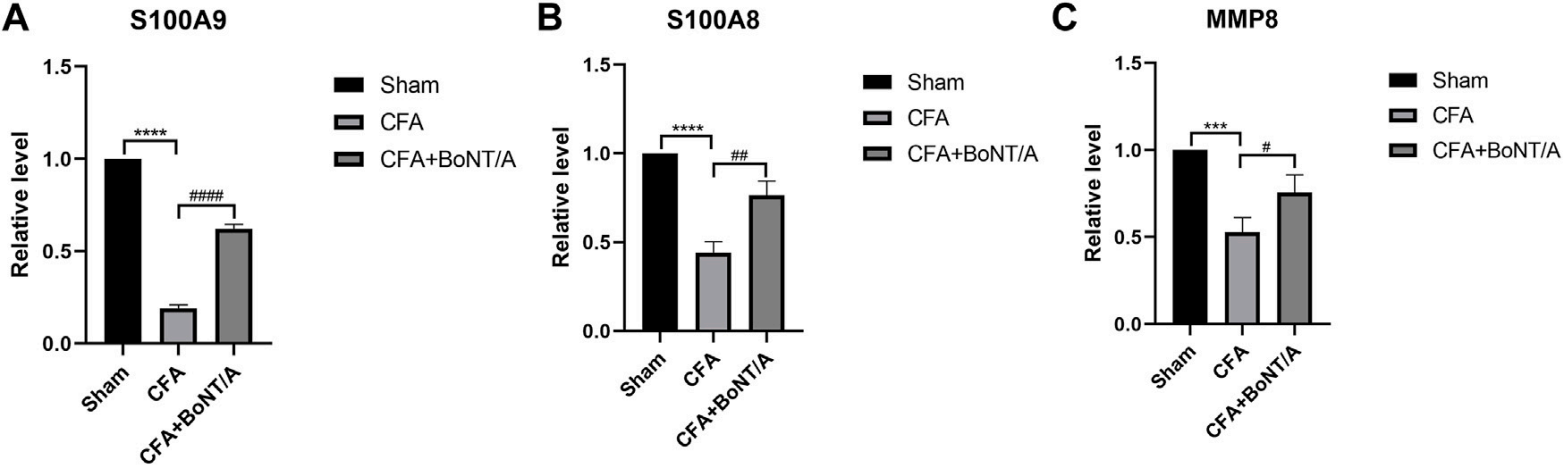
qRT-PCR results show that the expression levels of **(A)** S100A9, **(B)** S100A8, and **(C)** MMP8 were obviously lower after injection of CFA. After BoNT/A application, S100A9, S100A8, and MMP8 were obviously upregulated (****p < 0.0001, ***p < 0.001, CFA group vs. Sham group; ####*p* < 0.0001, ##*p* < 0.01, #*p* < 0.05, CFA+BoNT/A group vs. CFA group).

Three genes with high reliability and associated with inflammatory response were selected for verification. MCODE identified S100A9, S100A8, and MMP8 as the genes with higher score in cluster. We detected the expression of these genes in DRG by qRT-PCR. The results showed that the expression levels of S100A9, S100A8, and MMP8 in rats with arthritis pain were visibly lower than the Sham group. After injection of BoNT/A, S100A9, S100A8, and MMP8 showed high expression levels compared with the CFA rat model (Figure 6).

## 4 Discussion

Arthritis is the most common cause of chronic pain. In this study, chronic inflammatory pain induced by injection of CFA in rats. The previous study concluded that BoNT/A plays an important role in anti-inflammatory effects, but the exact anti-inflammatory effect of BoNT/A is not known (Wang et al., 2017). The current study investigated the analgesic effect of BoNT/A and revealed the anti-inflammatory effect of BoNT/A on arthritis and its mechanism by transcriptome analysis. We used RNA-seq to detect DEGs between the Sham group and the CFA group and between the CFA group and the CFA+ BoNT/A group. All of the DEGs were analyzed by GO, KEGG, and PPI network analysis and were verified by real-time PCR. These results showed the possible anti-inflammatory effect of BoNT/A in the CFA-induced arthritis model.

The results of pain-related behaviors showed that the PWT and PWL decreased significantly after CFA injection, and mechanical allodynia and thermal hyperalgesia were significantly reversed with administration of BoNT/A. Our previous study found that there was no difference in mechanical hyperalgesia and thermal hyperalgesia in rats after the injection of 3 and 10 U of BoNT/A; this result suggested that the antinociception of BoNT/A was not dose-dependent. We also found 5 days after BoNT/A injection that the anti-allodynic effects of BoNT/A was stable and did not improve over time (Fan et al., 2017). Therefore, the study chose 5 days after 3 U BoNT/A injection for the RNA sequencing.

In addition, the colocalization of cl-SNAP-25 in DRG indicates that the BoNT/A was transcytosed to DRG neuron cell. Antonucci et al. found the occurrence of cl-SNAP-25 in retina after injecting BoNT/A into the superior colliculus (Antonucci et al., 2008). Restani et al. reported that catalytically active BoNT/A is capable of undergoing transcytosis using the visual pathway as a model system in rat eye (Restani et al., 2011). These studies showed that BoNT/A is retrogradely transported from application sites to other regions. Our study suggested that BoNT/A can transport from peripheral application sites to DRG through retrograde axonal transport and reduce CFA-induced arthritic pain. Studies have shown that DRG neuron cells play a mediating role in arthritic pain, neuropathic pain, and inflammatory pain (Cobianchi et al., 2010; Svensson and Brodin, 2010; Vacca et al., 2012). Thus, a possible interpretation could be that BoNT/A transport from injection sites to DRG neuronal cells and may play an important role in antinociception.

GO classification and enrichment analysis revealed that CFA regulated various processes, such as response to lipopolysaccharide and inflammatory response. In addition, KEGG pathway analysis showed that CFA affected multiple signaling pathways, such as metabolic pathways, IL-17 signaling pathway, and NOD-like receptor signaling pathway. IL-17 signaling pathway plays a key role in a variety of inflammatory diseases such as psoriasis, rheumatoid arthritis, and psoriatic arthritis (Nirula et al., 2016). Data from experimental arthritis indicated IL-17 receptor signaling as a critical pathway in turning an acute synovitis into a chronic destructive arthritis (Lubberts, 2008). In the IL-17 signaling pathway, IL-17 changes the expression of inflammatory genes by inducing *de novo* gene transcription or stabilizing target mRNA transcription (Amatya et al., 2017). In addition, the IL-17 family signals through the corresponding receptors to activate downstream pathways including NF-κB, MAPKs, and C/EBPs, inducing the expression of antimicrobial peptides, cytokines, and chemokines, which amplified the inflammatory response (Gaffen, 2009; Beringer et al., 2016; Beringer and Miossec, 2019). The IL-17 signaling pathway has been an attractive target for the development of therapeutic agents to modulate aberrant inflammatory responses and many inhibitors have already been approved for the treatment of a range of inflammatory diseases (Hueber et al., 2010; Hueber et al., 2012). Finally, PPI network analysis of different genes in the CFA group and the Sham group indicated that there were 11 genes most associated with CFA-induced arthritis pain in rats. These genes encode protein about inflammatory response and might be the key genes of arthritis pain. These key genes might directly or indirectly affect the inflammatory response through different signaling pathways.

Furthermore, the mechanisms underlying the anti-inflammatory effect of BoNT/A against arthritis pain involved the PI3K–Akt signaling pathway, metabolic pathways, ECM–receptor interaction, focal adhesion, and cytokine–cytokine receptor interaction. Among them, the PI3K/Akt signaling pathway is considered to be an endogenous negative-feedback regulatory mechanism, which is activated by multiple types of cellular stimulation or toxic insults and regulates basic cellular functions, such as transcription, translation, proliferation, growth, and survival (Brugge et al., 2007; Carnero et al., 2008; Hers et al., 2011). Moreover, PI3K/Akt signaling has also been identified to affect several inflammatory-related regulators (Li et al., 2016; Pan et al., 2019). Guan et al. discovered that activation of the PI3K/Akt signaling pathway attenuates inflammatory reactions and myocardial apoptosis (Guan et al., 2020). Yu et al. demonstrated that the combination of minocycline (MC) and botulinum toxin (BoNT) significantly promoted the expression of SIRT1 both *in vivo* and *in vitro*, thereby inhibiting inflammatory and injury-related signaling pathways, such as NF-κB, p53, and PI3K/Akt (Yu et al., 2020). Su et al. systematically characterized the transcriptional responses of lung tissues for 72 h after aerosolized BoNT/A exposure; they found that the DEGs set IL-17, TNF-α, and NK-κB signaling pathways after 12 and 24 h of BoNT/A exposure, but there was an opposite change after 48 and 72 h of BoNT/A exposure, and the DEGs were enriched for anti-inflammation functions, such as regulation of inflammatory response. Therefore, they suggest that the changes in expression profiles in these experimental groups indicate a shift from a pro- to an anti-inflammatory phenotype in the distal lung parenchyma, and that BoNT/A might show an anti-inflammatory effect in this change (Su et al., 2021). Olex et al. found that another important signaling and metabolic pathway in the progression of osteoarthritis (OA) is the ECM–receptor interaction pathway (Olex et al., 2014). Koelling et al. also demonstrated that several dysregulated genes in OA samples were associated with ECM–receptor interactions and focal adhesions (Koelling et al., 2009); we speculate that these pathways might also be involved in the anti-inflammation effect, and BoNT/A might activate these signaling pathways, attenuate inflammatory response, and then exert analgesic effect.

In addition, PPI network analysis found that there were 11 highly correlated DEGs encoding inflammatory response proteins between the CFA group and the CFA+BoNT/A group. These DEGs might indicate the potential mechanism for the anti-inflammatory effect of BoNT/A on arthritis pain in rats. We found that the mRNA expression levels of S100A9, S100A8, and MMP8 were significantly decreased after CFA-induced arthritis pain, while BoNT/A increased the expression changes of these genes. Studies showed that two members of the S100 family, S100A8 and S100A9, are overexpressed at local sites of inflammation, which have been proven to be useful genes of inflammatory diseases such as arthritis, bowel disease, and chronic inflammatory lung (Foell et al., 2004). However, an anti-inflammatory effect of the S100A8/S100A9 complex was reported in the rat with adjuvant-induced arthritis. Other studies reported that neutrophils and the calcium-binding protein S100A9 are involved in the control of inflammatory pain (Pagano et al., 2002; Pagano et al., 2006). Transcriptional changes of the S100 family occurred in adult skeletal muscle after BoNT/A treatment (Mukund et al., 2014). Several studies have shown that MMP8, also known as neutrophilic collagenase, is associated with neuroinflammatory diseases such as bacterial meningitis, spinal cord injury, and multiple sclerosis (Kuula et al., 2009). MMP8 plays an anti-inflammatory role in host defense by treating anti-inflammatory cytokines and chemokines (Owen et al., 2004). In addition to regulating apoptosis and immune response, MMP8 also plays a protective role in lung inflammation (Gueders et al., 2005), cancer progression (Balbin et al., 2003; Gutierrez-Fernandez et al., 2008; Korpi et al., 2008), and wound healing (Gutierrez-Fernandez et al., 2007). The present study found that S100A9, S100A8, and MMP8 were downregulated in DRG after CFA injection and upregulated after BoNT/A injection. For this result, we speculated that BoNT/A might regulate key anti-inflammatory genes to attenuate inflammatory pain.

The DEGs were analyzed by different tools; for the results of analysis, both signaling pathways and key genes pointed to the anti-inflammatory effect of BoNT/A, which proves that BoNT/A can achieve the purpose of analgesia by exerting an anti-inflammatory effect. Interestingly, the study confirms our previous inference at the genetic level. The possible interpretation is that BoNT/A can regulate key anti-inflammatory genes and then activate the signaling pathways to amplify and exert an analgesic effect. However, it should be acknowledged that there are limitations inherent to the current study design and that several questions remain open. qRT-PCR is not the best way to verify the current conclusion, so the next study will validate the correlation between expression levels of mRNA and proteins to further support our conclusion in the future. The relationship between the three key genes and the signaling pathways and how to affect the signaling pathways to play an anti-inflammatory role still need to be further explored.

In summary, the current study identified three key genes that are closely associated with the effect of BoNT/A against CFA-induced arthritis pain. In addition, molecules significantly associated with some pathways were found to be important for analgesia of BoNT/A. The identification of these genes can provide new therapeutic targets for inflammatory pain and affect the signaling pathways after the treatment of BoNT/A.

## Data Availability

The datasets presented in this study can be found in online repositories. The names of the repository/repositories and accession number(s) can be found below: https://www.ncbi.nlm.nih.gov/sra/PRJNA741696.
